# The effect of human resource management on performance in hospitals in Sub-Saharan Africa: a systematic literature review

**DOI:** 10.1186/s12960-018-0298-4

**Published:** 2018-08-02

**Authors:** Philipos Petros Gile, Martina Buljac-Samardzic, Joris Van De Klundert

**Affiliations:** 1 Higher Education Institutions’ Partnership, PO BOX 14051, Addis Ababa, Ethiopia; 2 Erasmus School of Health Policy & Management, Erasmus University Rotterdam, PO Box 1738, 3000 DR Rotterdam, The Netherlands; 3 Prince Mohammad Bin Salman College (MBSC) of Business & Entrepreneurship, 7082-BayLaSun-Juman St. Unit No. 1, King Abdullah Economic City, 23964-2522 Kingdom of Saudi Arabia

**Keywords:** Systematic review, HRM, SSA, Hospital, Performance, Outcomes, Health workforce

## Abstract

Hospitals in Sub-Saharan Africa (SSA) face major workforce challenges while having to deal with extraordinary high burdens of disease. The effectiveness of human resource management (HRM) is therefore of particular interest for these SSA hospitals. While, in general, the relationship between HRM and hospital performance is extensively investigated, most of the underlying empirical evidence is from western countries and may have limited validity in SSA. Evidence on this relationship for SSA hospitals is scarce and scattered. We present a systematic review of empirical studies investigating the relationship between HRM and performance in SSA hospitals.

Following the PRISMA protocol, searching in seven databases (i.e., Embase, MEDLINE, Web of Science, Cochrane, PubMed, CINAHL, Google Scholar) yielded 2252 hits and a total of 111 included studies that represent 19 out of 48 SSA countries.

From a HRM perspective, most studies researched HRM bundles that combined practices from motivation-enhancing, skills-enhancing, and empowerment-enhancing domains. Motivation-enhancing practices were most frequently researched, followed by skills-enhancing practices and empowerment-enhancing practices. Few studies focused on single HRM practices (instead of bundles). Training and education were the most researched single practices, followed by task shifting.

From a performance perspective, our review reveals that employee outcomes and organizational outcomes are frequently researched, whereas team outcomes and patient outcomes are significantly less researched. Most studies report HRM interventions to have positively impacted performance in one way or another. As researchers have studied a wide variety of (bundled) interventions and outcomes, our analysis does not allow to present a structured set of effective one-to-one relationships between specific HRM interventions and performance measures. Instead, we find that specific outcome improvements can be accomplished by different HRM interventions and conversely that similar HRM interventions are reported to affect different outcome measures.

In view of the high burden of disease, our review identified remarkable little evidence on the relationship between HRM and patient outcomes. Moreover, the presented evidence often fails to provide contextual characteristics which are likely to induce variety in the performance effects of HRM interventions. Coordinated research efforts to advance the evidence base are called for.

**Electronic supplementary material:**

The online version of this article (10.1186/s12960-018-0298-4) contains supplementary material, which is available to authorized users.

## Background

While Sub-Saharan Africa (SSA) is home to 12% of the global population [1], it employs 3.5% of the global health workforce to service a disproportionate 27% of the global burden of disease [2]. A majority of countries across the globe for which the health workforce shortage is classified as critical (36 out of 57) lie in SSA [3, 4]. Most SSA countries are not able to attain an average health workforce density of 2.5 per 1000 population as recommended by the World Health Organization (WHO) [5, 6] and half of the SSA countries have fewer than ten physicians per 100,000 people (while Western countries commonly have 250 per 100,000 or more) [5, 7–9]. The low workforce density and high workload in SSA especially impacts hospital [6, 7]. The shortage of supply to match demand further increases because of low retention rates among skilled health workers [8–12]. Implementation of human resource management (HRM) practices is needed to improve the situation for a depleted and overstretched health workforce, and patient outcomes [10, 13–18].

Research on HRM interventions in SSA hospitals have so far primarily addressed (human) resource availability, e.g., “head counts,” technical skills, and basic working conditions [19–28]. These practices are often referred to as “hard” HRM [29]. Hard HRM refers to approaching employees as one of several categories of organization resources (e.g., financial resources, equipment) that influence organizational effectiveness and are mostly organization-centered and reactive [26, 29, 30]. Although hard HRM practices have shown to be related to improved performance outcomes (e.g., waiting time, quality of care, patient experiences) [18, 31, 32], broader HRM interventions are needed to sustain hospital service quality and retain a satisfied workforce [10, 24].

Soft HRM practices are more employee-centered and focused on work-environment. They single out human resources as most important and subsequently address training and development needs, tasks and roles, communication, delegation, and motivation [29, 33, 34]. In the last decade, especially soft HRM practices have shown to impact performance, sometimes in combination with hard HRM practices [25, 33, 35]. However, understanding and the adoption of soft HRM practices in SSA hospitals is limited [18, 36–38].

The growing evidence of the relationship between HRM practices and performance has shown to be complex and is frequently referred to as “black box” [39–42]. Dieleman et al. underline the importance of context when stating that a HRM practice may result in different outcomes when applied in different contexts, as contextual factors are likely to influence outcomes [16].

The current evidence base on effectiveness of HRM practices is mainly developed in particular research settings, namely hospitals in the USA and Western Europe. Next to the high variation within these settings (e.g., type of hospital, financial management, government), there are major differences compared to the SSA setting (e.g., low providers capacity, low economic status, challenging socio-cultural issues, demographic trends, high disease burden). It is therefore likely to have limited validity in SSA [34]. A first relevant and major contextual difference is formed by the combination of a disproportionally high burden of disease and health workforce shortages occurring in SSA contexts, which so explicitly outline the societal relevance of understanding the relationship between HRM practices and performance [43–46]. In addition, major cultural differences exist, as well as differences in public service infrastructures and operations [36], financial resource limitations, availability and quality of medicines, materials and equipment, disease prevalence, and health literacy [10, 34, 37, 47–52]. Rowe et al. highlighted the need to generate knowledge about the strategies to improve performance by HRM practices in low-resource settings and called for dedicated and updated systematic reviews [18]. Harries and Salaniponi underlined this by stating that “getting the most out of the already depleted and overstretched health workforce in resource-poor areas is a priority” [52]. This study presents a systematic literature review on the relationship between HRM and performance for SSA hospitals.

## Methods

We conducted this systematic literature review following the Preferred Reporting Items for Systematic Reviews and Meta-Analyses (PRISMA) [53–55].

### Search strategy

The search included seven databases (see Table 1) with search terms from three categories:

1. The geographical SSA setting as defined by United Nations [56]. For example, terms regarding SSA or the SSA countries separately (e.g., Benin, Ethiopia, Kenya, South Africa).
2. Healthcare setting and healthcare workforce. For example, hospitals or physician.
3. Terminologies related to HRM practices. For example, human resource management, training, skills, motivation, competences, or compensation.

**Table 1:** Number of hits per database

Database	Number of hits
Embase	1 217
MEDLINE	355
Web of Science	186
Cochrane	1
PubMed	49
CINAHL	286
Google Scholar	157
Total	2 251

Additional file 1 provides search term details. The search strategy was conducted in collaboration with a librarian from a medical library specialized in designing systematic reviews in April 2016. The search strategy resulted in 2251 titles/abstracts (doubles excluded) (see Table 1).

### Inclusion/exclusion criteria

Studies were included if they met the following inclusion criteria: (1) Empirical study, regardless of the research methods; (2) focusing on links between HRM and performance outcomes; (3) SSA region; (4) hospital setting; (5) English language; and (6) published in a peer reviewed scientific journal.

Studies were excluded based on the following exclusion criteria: (1) focus on technical skills only (e.g., clinical skills training) as opposed to non-technical skills (e.g., team work training, personal communication training) [57, 58]; (2) HRM interventions which were not under the control of hospital management but enforced by the Ministry of Health or external partner organizations such as the WHO (e.g., a national HIV educational intervention); and (3) studies that solely address capacity shortage (e.g., employing additional nurses). Studies which solely report on reducing capacity shortages are excluded as they are expected to improve effectiveness by definition.

### Selection strategy

**Fig. 1:**
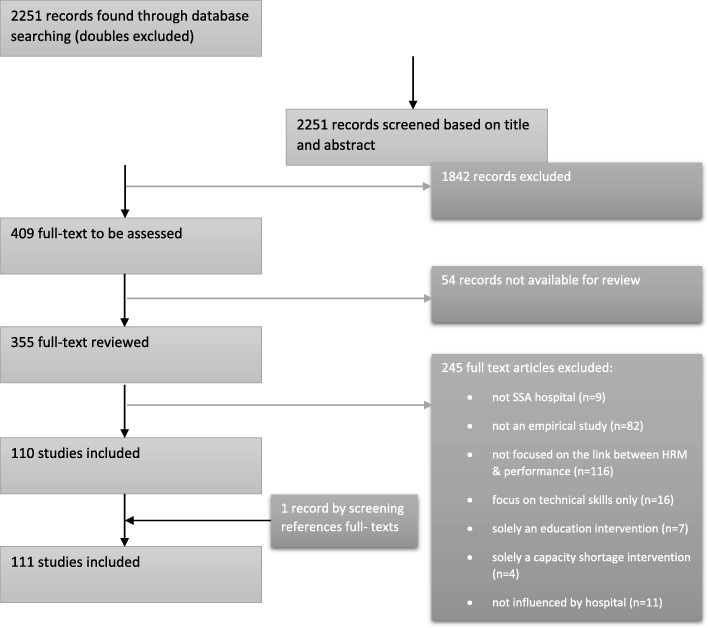
PRISMA Flow Diagram

1. We followed a four-stage selection process using a structured Excel format [59]: screening the title and abstract on the in- and exclusion criteria. This was performed independently by two authors. In case of disagreement between the two authors, the third author decided or postponed the decision to the next stage. The first stage reduced the initial search of 2251 hits to 409 hits.
2. Examining the full text on the in- and exclusion criteria. The second stage was also performed by two authors. In case of disagreement, the third author was included to make the final decision. The second stage reduced the publications to 110 articles.
3. Summarizing all accepted full articles by the first author.
4. Reference and biography check of the summarized articles resulted in including one additional article and hence a total of 111 included articles (see Fig. 1).

### Data analysis

The first data analysis step was to collect all HRM practice and all performance outcomes from the included studies. These “raw” practices and outcomes were discussed within the research team and processed iteratively to determine common “labels” for the practices and outcomes. These labels practices and outcomes where subsequently structured in categories. Building on previous syntheses in HRM effectiveness research [19, 20, 27, 60], we distinguished five categories of (single) HRM practices (see Table 5):

1. Training and education;
2. Salary and compensation;
3. Rostering and scheduling;
4. Task shifting; and
5. Managing employees (through leadership support and mentoring).

All labeled practices from the data collection process on single HRM practices were categorized accordingly. Additional file 2 presents the number of studies that link a specific HRM practice to a specific outcome. Studies presenting research on *HRM bundles*, i.e., interventions which combine multiple practices, are classified following Subramony ([28], p. 746-747]) (see Table 2). The five categories of single HRM practices can be placed under the classification of Subramony as follows: empowerment enhancing (task shifting), motivation enhancing (salary and compensation, rostering and scheduling, managing employees), and skills enhancing (training and education).

**Table 2:** Content of HRM bundles according to Subramony (2009)

*Empowerment-enhancing bundles* Employee involvement in influencing work process/outcomes Formal grievance procedure and complaint resolution systems Job enrichment (skill flexibility, job variety, responsibility) Self-managed or autonomous work groups Employee participation in decision making Systems to encourage feedback from employees	
*Motivation-enhancing bundles* Formal performance appraisal process Incentive plans (bonuses, profit-sharing, gain-sharing plans) Linking pay to performance Opportunities for internal career mobility and promotions Health care and other employee benefits	
*Skills-enhancing bundles* Job descriptions/requirements generated through job analysis Job-based skill training Recruiting to ensure availability of large applicant pools Structured and validated tools/procedures for personnel selection	

The *performance outcome* dimensions were categorized into four categories:

1. Employee outcomes (employee performance, job satisfaction, turnover intention or retention, motivation, workload reduction, reduction of moonlighting);
2. Team performance outcome;
3. Organizational outcomes (quality of care, waiting time, efficiency, patient safety/error reduction, staff shortage reduction); and
4. Patient outcomes (patient experience, clinical outcome).

### Quality appraisal

We appraised the quality of the studies using the revised version (2011) of the Mixed Methods Appraisal Tool (MMAT) [61–63], as commonly applied in systematic reviews (e.g., [64–67]). For qualitative and quantitative studies, the scores represent the number of criteria met, varying from one criterion met (*) to all criteria met (****). For mixed method studies, the scores represent the lowest score of the quantitative and qualitative components, as the quality of the study cannot surpass the quality of its weakest component. Tables 5 and 6 present the MMAT scores of the included studies.

## Results

### Study characteristics

The selected studies represent 19 out of 48 SSA countries (presented in Additional file 3). The six most studied countries are South Africa (32 studies), Tanzania (14), Kenya (13), Nigeria (10), Ethiopia (8), and Uganda (8). Five studies researched hospitals in multiple SSA countries. As a research setting, 16 studies simply mention hospitals without specifying the type of hospital, in contrast to the others that specified whether it regarded public, national, private, missionary, teaching, district, secondary care, rural, and/or primary care hospitals. The research included 36 qualitative (32.4%), 57 quantitative (51.3%), and 18 mixed methods (16.2%) studies. Table 3 displays the MMAT quality scores of the included studies.

**Table 3:** Number of studies with study design and methodological appraisal scores

MMAT score					
Study design	25%	50%	75%	100%	Total
	*	**	***	****	
Quantitative	–	13	18	26	57
Qualitative	1	8	13	14	36
Mixed methods	1	3	9	5	18
Total	2	24	40	45	111

### Link between HRM practices and performance outcomes

Table 4 shows that while most studies (*n* = 85, 76.6%) considered a bundle of HRM interventions (as opposed to a single practice intervention), they typically addressed only one performance outcome (*n* = 81, 73.0%). For ease of exposition, we now first present a narrative synthesis of the results on single HRM practices and subsequently of the results on HRM bundles. Table 5 (single HRM practices) and Table 6 (HRM bundles) present detailed review findings and the corresponding references.

**Table 4:** Number of performance outcomes for HRM practices

HRM practices	Number of performance outcomes			Total
	1 outcome	2 outcomes	3 outcomes	
Single HRM practice	19	7	0	26
Bundles of HRM practices	62	22	1	85
Total number of studies	81	29	1	111

**Table 5:** Overview of single HRM practices in relation to performance outcomes

Authors, year, country	HRM practices	Employee outcome	Team outcome	Organizational outcome	Patient outcome	MMAT Score
1. Training and education						
Ajayi, 2013, Nigeria [75]	training nurses on computer-skills			improved efficiency	–	***
Eygelaar & Stellenberg, 2012, S.Africa [94]	training on nursing care			improved quality of care	–	****
Issahaku et al., 2012, Ghana [100]	training (clinical and administrative staff)	improved performance			–	****
Jacobs & Roodt, 2008, S.Africa [102]	knowledge sharing organizational culture /learning practice among professional nurses	reduced turnover intention			–	****
Esan et al., 2014, Nigeria [148]	training residence doctors	improved job satisfaction			–	***
Letlape et al., 2014, S.Africa [150]	in-service training on confidence building			improved quality of care	–	**
Mduma et al., 2015, Tanzania [155]	simulation training on delivery and neonatal care				decreased mortality	**
Bergman et al., 2008, Tanzania [146]	trauma team training of physicians and nurses	improved job satisfaction	improved team performance		–	*
Uys et al., 2005, S.Africa [166]	training on supportive supervision	improved job satisfaction		improved quality of care	–	**
Crofts et al., 2015, Zimbabwe [172]	onsite-team training on obstetric emergency care		improved team performance in clinical practices		improved maternal deaths	**
2. Salary and compensation						
Aberese-Ako et al., 2014, Ghana [69]	incentives /monthly transport allowances	improved performance			–	****
Nwude & Uduji, 2013, Nigeria [120]	fair and adequate compensation	improved job performance			–	**
Atambo et al., 2013, Kenya [83]	implementing incentive systems	improved performance		improved efficiency of service delivery	–	***
Ashmore & Gilson, 2015, S. Africa [80]	additional wage incentives for specialists	improved retention			–	****
3. Rostering and scheduling						
McIntosh & Stellenberg, 2009, S. Africa [154]	implementing staff control strategy/scheduling/ to control moonlighting	turnover intention continued (not improved)		improved quality of care	–	**
Nyathi & Jooste, 2008, S. Africa [121]	managing reutilization and workload	reduced absenteeism among nurses			–	***
Osisioma et al., 2015, Nigeria [122]	implementation of flexible working arrangements	improved performance			–	**
Rispel et al., 2014, S.Africa [126]	managing rostering & scheduling to control moonlighting	reduced intention to leave			–	****
4. Task shifting						
Ferrinho et al., 2015, Mozambique & Zambia [95]	task shifting practice			reduced staff shortage and improved quality of care	–	***
Jennings et al., 2011, Benin [103]	task shifting practices for lay nurse aides			improved efficiency of health care	–	**
Olson et al., 2014, Malawi [161]	task shifting in patient triage and treatment			improved quality of care	reduced inpatient mortality	***
Sanjana et al., 2009, Zamia [164]	task shifting for lay counselors			reduced staff shortage, reduced rate of errors and	–	**
Galukande et al., 2013, Uganda [96]	task shifting (surgical) practice			improved staff shortage	decreased mortality	****
O’Malley et al., 2014, Namibia [162]	task shifting from doctors to nurses			improved quality of service	–	***
5. Managing employees						
Nigussie & Demissie, 2013, Ethiopia [158]	leadership styles of nurse managers	increased job satisfaction			–	****
Okurame, 2009, Nigeria [160]	mentoring practices	improved job satisfaction			–	****

**Table 6:** Overview of HRM bundles in relation to performance outcomes

Author, year, country	HRM themes			Performance outcomes			MMAT Score
	Empowerment -Enhancing practices	Motivation-Enhancing practices	Skills-Enhancing practices	Employee outcome	Organizational outcome	Patient Outcome	
Ajemigbitse et al., 2013, Nigeria [68]	supportive supervision		job-based skill training		improved prescribing errors among junior physicians		***
Ackerman & Phil, 2007, S.Africa [70]	teamwork	management support, scheduling		improved job satisfaction			**
Francis & Roger, 2012, Ghana [71]	supervision	salary, supplementary allowances, leadership support, recognition	job-based skill training	improved retention and staff motivation			****
Simiyu & Moronge, 2015, Kenya [72]	teamwork, work-life balance, communication practice	salary, supplementary benefits	recruitment	improved performance			****
Allegrazi et al., 2010, Mali [73]	feedback on performance		training		improved patient safety		****
Akinyemi & Atilola, 2013, Nigeria [74]		salaries	training	improved job satisfaction			****
Abubeker et al., 2014, Nigeria [76]		compensation	training	reduced turnover intention			***
Asegid et al., 2014, Ethiopia [77]		salary, supplementary allowances	training	improved job satisfaction and reduced intention to leave			****
Ackerman & Bezuidenhout, 2007, S.Africa [78]	teamwork	scheduling(flexi-time system)		staff turnover(continued)			***
Ashmore, 2013, S.Africa [79]		supplementary allowances	job-based skill training	improved job satisfaction and reduced moonlighting			****
Nyakundit et al., 2012, Kenya [81]		recognition, incentives	training	improved performance	improved quality of care		***
Atambo et al., 2013, Kenya [82]		recognition, incentives	training	improved performance	improved efficiency in service delivery		****
Aveling et al., 2015, Rwanda & Ethiopia [84]	teamwork		training, staffing		improved quality of care and safety of care		****
Awasses et al., 2013, Namibia [85]		recognition, staff performance appraisal, remuneration, supplementary financial allowances	in-service training	improved performance of nurses			***
Ayeiko et al., 2011, Kenya [86]	supervision, feedback		training		improved quality of care		****
Waju et al.,2011, Ethiopia [87]		management support	staffing, training	improved performance		improved patient satisfaction	****
Bhengu, 2000, S. Africa [88]		rostering & scheduling, salary		reduced intention to leave improved motivation			****
Bradley et al., 2008, Ethiopia [89]			mentorship, training		improvement in hospital management skills (efficiency)		****
Bradley & McAuliffe, 2009, Malawi [90]		remuneration	training	improved performance improved staff retention			***
Dagne et al., 2015, Ethiopia [91]	communication/supervisor feedback	job content management of schedules, performance review, financial incentives, recognition	staffing	improved motivation of health professionals	improved quality of care		****
De Brouwere et al., 2009, Senegal [92]	teamwork, task shifting					improved maternal mortality	***
Dieleman et al., 2006, Mali [93]		salary, performance appraisal, reward system	training	improved motivation			****
Hall, 2004, S. Africa [97]		salary, supplementary incentives/ allowances, scheduling		reduced intention to leave			**
Honda & Vio, 2015, Mozambique [98]		incentives, scheduling, salaries	job-based skill training	improved job satisfaction and retention			***
Libeziako et al., 2013, S. Africa [99]	teamwork practice	salary, supplementary allowances		improved motivation			**
Jack, 2013, Ghana [101]	teamwork	compensation, allowances	recruitment/staffing, training	improved retention			**
Kamanzi & Nikosi, 2011, Rwanda [104]		remuneration, recognition	job-based skill training	improved level of motivation			****
Kekana et al., 2007, S.Africa [105]	teamwork	performance appraisal, remuneration, scheduling		improved job satisfaction			****
Khamis & Njau, 2014, Tanzania [106]		salary, allowances, management support, rostering & scheduling	staffing, training		improved quality of care at outpatient		****
Kotzee & Couper, 2006, S. Africa [107]		salaries, allowances, recognition	training, mentorship	improved retention of doctors			****
Kruger & Bezuidenhout, 2015, S. Africa [108]		scheduling, promotion, management support	training	reduced female doctors dissatisfaction in balancing professional work and family lives			****
Liphoko et al., 2006, S.Africa [109]		performance appraisal, promotion, management support	job-based skill training	improved job satisfaction of nurses			***
Leshabari et al., 2008, Tanzania [110]	communication/ feedback	performance evaluation, salaries		improved job satisfaction and motivation			****
Longmore & Ronnie, 2014, S.Africa [111]	communication	salaries, performance appraisal	training	improved retention of doctors			*
Luboga et al., 2011, Uganda [112]		compensation/salaries, benefits, recognition, scheduling workload, management support	training, staffing	improved job satisfaction and retention of physicians			**
Makapela & Useh, 2015, S.Africa [113]		salary, management support, allowance	job-based skill training	improved retention			***
Mathauer & Imhoff, 2006, Benin & Kenya [114]	supervision	recognition, allowances, salary	job-based skill training	improved motivation			****
Mbindyo et al., 2009, Kenya [115]	employee engagement, communication	promotion, leadership support, performance appraisal, incentives			improved quality of care		****
McAuliffe et al., 2009, Malawi [116]	teamwork	management support	staffing	improved task performance			**
McAuliffe et al., 2013, Malawi, Tanzania & Mozambique [117]	job autonomy, task shifting, teamwork, supervision	leadership support		improved job satisfaction and reduced intention to leave			***
Mokoka et al., 2010, S.Africa [118]		salary, rostering & scheduling, management support	training	improved retention of nurses			***
Mubyazi et al., 2012, Tanzania [119]	supervision	Incentives	staffing, training	improved motivation			***
Pieterson, 2005, S.Africa [123]		pay, management support, scheduling, promotion		improved job satisfaction			***
Pillay, 2009, S.Africa [124]	teamwork, job autonomy, job security	rostering & scheduling	training	improved job satisfaction and motivation			****
Prytherch et al., 2012, Tanzania [125]		rostering & scheduling, salaries, incentives, recognition/promotion		increased job performance			***
Selebi & Minnaar, 2007, S.Africa [127]	supportive supervision	salaries		improved job satisfaction			***
Sikwese et al., 2010, Zambia [128]			staffing/ selection, training		improved efficiency of service delivery		***
Siril et al., 2011, Tanzania [129]	supervision, teamwork	compensation, rostering & scheduling	training		improved quality of care		****
Ssengooba et al., 2002, Uganda [130]		rostering & scheduling	staffing		improved hospital performance (efficiency and effectiveness)		**
Stodel & Stewart-Smith, 2011, S.Africa [131]	supervision	scheduling	training, mentorship	improved retention			***
Tabatabai et al., 2013, Tanzania [132]	employee engagement	salary, incentives, scheduling, management support	training	reduced internal migration (public to private)			**
Thatte & Choi, 2014, Kenya [133]	supervision		written job descriptions, training		improved service quality		**
Uwaliraye et al., 2013, Rwanda [134]	feedback		training	improved performance of nurses and midwives			***
Yami et al., 2011, Ethiopia [135]	teamwork	supplementary allowances, salary	training	improved job satisfaction			****
Bekker et al., 2015, S. Africa [136]	communication	rostering & scheduling		enhanced job satisfaction			***
Chandler et al., 2009, Tanzania [137]		salary, management support, rostering & scheduling	training	improved performance	improved quality of care		****
Chi et al., 2015, Burundi & Uganda [138]		rostering & scheduling, remuneration	staffing		improved quality of maternal care		***
Chirwa, 2000, Malawi [139]		performance appraisal	Staffing		improved quality of care		***
Hollup, 2012, Mauritius [140]	job security and safety	salary		improved staff motivation			****
Klopper et al., 2012, S.Africa [141]		wages, study leave opportunities	skills-training for career advancement	job dissatisfaction			**
Lasebitan & Oyetundt, 2012, Nigeria [142]		rostering & scheduling, wages	staffing	improved retention			****
Mudaly & Nkosi, 2015, S.Africa [143]		scheduling, promotion, pay, rewards/incentives	training, staffing	reduced absenteeism			***
Tibandebage et al., 2015, Tanzania [144]	supervision	incentives, salaries, leadership support, rostering & scheduling	Staffing	improved performance			***
Courtright et al., 2007, Malawi, Uganda, Tanzania & Kenya [145]	supervision	management support	Training	improved performance			****
Doherty et al., 2013, S.Africa [147]	supervision, task shifting				improved quality of care, reduced staff shortage and workload		***
Kamau & Omondi, 2015, Kenya [149]		supplementary allowances/incentives	job-based skill training	improved staff retention			****
Madzimbamuto et al., 2014, Botswana [151]	supervision		Training		improved quality of care		**
Mahlo & Muller, 2000, S.Africa [152]	communication		Training		improved quality of care		****
Manongi et al., 2009, Tanzania [153]		Salary	Training	improved performance			***
Nabirye, 2010, Uganda [156]		scheduling, pay, incentives/allowances		improved performance of nurses and job satisfaction			***
Ndetei et al., 2008, Kenya [157]		Salary	Training	reduced migration of health workforce (retention)			***
Okeke, 2008, Nigeria [159]		salary	Recruitment	improved retention			**
Rauf et al., 2008, S.Africa [163]	task shifting	scheduling, performance evaluation/appraisal			reduced waiting time (maximized efficiency)		**
Thomas & Valli, 2006, S.Africa [165]		scheduling, salary	training, staffing	improved job satisfaction			****
Yeboha et al., 2014, Ghana [167]	communication	management support	Training	improved retention			***
Rawlins et al., 2003, Kenya [168]	feedback, teamwork	management support	staffing, written job descriptions		improved organizational performance (efficiency)		***
Giuseppe et al., 2002, Kenya [169]	communication, work-life balance	scheduling, management support	Training	improved task performance and improved retention of resident doctors			***
Ngao, 2013, Kenya [170]			recruitment/staffing, training, mentorship		improved quality of care		**
Kotagal et al., 2009, Rwanda [171]		leadership support	staffing			improved patient satisfaction	**
Dowing, 2016, Uganda [173]			training, mentorship	improved nurses’ performance			***
Faye et al., 2013, Senegal & Mali [174]		salary, supplementary allowances, scheduling, management support	training	improved job satisfaction			****
Doef et al., 2011, Kenya, Tanzania & Uganda [175]		scheduling, management support, supplementary allowances	staffing	improved job satisfaction and reduced level of burnout			****
Srofenyoh et al., 2012, Ghana [176]	teamwork, communication	leadership support	training	improved employee performance		improved patient satisfaction and clinical outcomes	***
Woldegabriel et al., 2016, Ethiopia [177]	communication	scheduling, performance appraisal	selection/recruitment, training	improved intrinsic motivation of health workforce			****
Puoane et al., 2008, S.Africa [178]	teamwork, supervision, feedback	leadership support, monitoring performance	in-service training and induction of new nurses	improved task performance	improved quality of care in the better performing hospitals		****

### Single HRM practices and performance outcomes

In total 18 single HRM practices were researched (see Additional file 2). The single HRM practices are clustered in five categories:

#### (1) Training and education

Ten studies presented evidence on the relationship between training and outcomes [75, 94, 100, 102, 148, 150, 155, 146, 166, 172]. Six of these studies considered employee outcomes, two of which found a positive association with job satisfaction and retention [166, 172]. Four other studies (from South Africa, Tanzania, and Nigeria) [94, 102, 146, 148] found negative relationships between training and employee outcomes. Two of these studies also reported improved team performance as a result of team-training, but their evidence was qualified as weak [146, 172]. Three of the four studies focusing on organizational outcomes reported improvements in the quality of care [94, 150, 166]. The two studies reporting on patient outcomes found non-significant reductions in (maternal) mortality rates [155, 172].

#### (2)Salary and compensation

Research on salary and compensation almost exclusively regarded individual employee level outcomes (four studies). More specifically, they reported employee performance improvement [69, 83, 120], and one study reported improved employee retention [80].

#### (3)Rostering and scheduling

The four studies on rostering and scheduling each reported different, yet positive, effects on employee outcomes or organizational outcomes [121, 122, 126, 154]. One low-quality study [154] reported failure of HRM interventions (e.g., staff control strategies and scheduling/rostering) to reduce turnover intention.

#### (4)Task shifting

The six studies that researched task shifting/task delegation reported organizational outcomes. Three of the studies reported improvement in efficiency, while the other three reported to have reduced employee shortages. Interestingly, the evidence reported on the relationship with clinical outcome and quality of care was inconclusive (e.g., [95, 96]).

#### (5)Managing employees through leadership support and mentoring

The two studies which involved leadership and mentoring practices both reported improved job satisfaction by employees [158, 160].

### HRM bundles and performance outcomes

Table 6 shows that the majority of the studies that researched HRM bundles have considered bundles that combine practices from multiple HRM themes (i.e., empowerment, motivation, and skill).

#### Motivation-enhancing HRM practices

Motivation-enhancing practices (*n* = 71, 83.5%) are the most researched in SSA and refer to intrinsic and/or extrinsic motivation-enhancing HRM practices in a bundle. Five studies (6%) considered bundles that only included motivation-enhancing practices [88, 97, 123, 125, 156]. These studies reported improved employee outcomes, such as job satisfaction, performance, retention, and staff motivation.

Many studies reported on bundles combining motivation- and skills-enhancing practices (*n =* 34, 40%). (e.g. [74, 77, 85, 90, 93, 104, 106–108, 113, 118, 130, 131, 138, 139, 142, 143, 149, 153, 157, 159, 165, 174]). These bundles are mainly linked to positive employee outcomes (e.g., improved job satisfaction, retention, and performance) and to a lesser extent to organizational outcomes. Notable is that two studies [112, 141] showed inconclusive relationships with job satisfaction and staff retention.

Eleven studies (12.9 %) considered bundles which combined motivation-enhancing and empowerment enhancing HRM practices [70, 78, 99, 105, 110, 115, 117, 127, 136, 140, 163]. These bundles were largely linked to employee outcomes (e.g., improved job satisfaction, motivation) and to a lesser extent to organizational outcomes. Notable, one study reported how a bundle which combined empowerment-enhancing (team work) and motivation-enhancing HRM interventions (flexi-time system, scheduling) failed to reduce staff turnover [78].

Twenty-one studies (24.7%) utilized practices from each of the three categories empowerment-, motivation-, and skills-enhancing HRM practices. The results in these studies again mostly present improved employee outcomes (e.g., task performance, retention, motivation, and satisfaction) and some present improved organizational outcomes (e.g., quality of care and efficiency).

The majority of the studies included extrinsic motivation practices, such as salary (*n* = 40, 47.1%) and supplementary allowances/incentives (*n =* 27, 31.8%). These financially oriented incentives were most frequently combined with the skills enhancement intervention training (32 studies), and less with empowerment interventions (13 studies). Six studies reported a combination of financial incentives with teamwork [72, 99, 101, 105, 129, 135], and six studies with supervision [71, 114, 119, 127, 129, 144]. In general, these studies reported significant and positive effects on the employee outcomes job satisfaction (13 studies), employee retention (8 studies), and employee performance (9 studies). Two studies, however [78, 111], reported non-significant effects on employee retention, and one study reports a negative effect on job satisfaction [141]. Only three of these financial incentive-related studies reported on organizational performance (i.e., quality of care) [86, 115, 129].

Scheduling and rostering were also frequently reported motivation-enhancing practices (*n =* 31, 36.5%). Scheduling and rostering were often combined with skills-enhancement interventions (18 studies) and empowerment-enhancing practices (13 studies). Of these studies, 23 reported positive effects on the employee outcomes turnover intention, job satisfaction, and/or employee performance. Positive effects on the organizational outcomes quality of care and reduced waiting time were reported by eight studies.

Leadership/management support practices (*n =* 24, 28.2%) were researched mostly in combination with the skills-enhancing interventions training and staffing, along with the empowerment-enhancing practices team work and supervision. In general, these studies reported significant improvement and positive effects on employee outcomes (e.g., staff retention, job satisfaction and task performance), organizational (e.g., quality of care), and patient outcomes. Some studies [112, 116, 171] showed inconclusive results on the relationships with employee outcomes and patient satisfaction.

Less frequently researched were bundles using motivation-enhancing practices based on recognition (*n* = 16, 18.8%) and staff performance appraisal (*n =* 12, 14.1%), which have often been combined with skills-enhancing training and empowerment-enhancing practices (e.g., task shifting, communication, team work, employee engagement). These studies reported significant improvements and positive effects on employee outcomes (e.g., performance, retention, job satisfaction and intrinsic motivation) and organizational outcomes (e.g., quality of care, reduced waiting time).

#### Skills-enhancing HRM practices

Skills-enhancing HRM practices were researched in 66 studies (77.6%). These studies mostly focused on training, staffing, and mentorship. Only four studies (4.7%) researched bundles that solely contained skills-enhancing practices [89, 128, 170, 173]. Three of these studies showed significant improvements in organizational outcomes (e.g., efficacy and quality of care) [89, 128, 170] while one study reported enhanced employee performance [173]. As a side effect, some studies mentioned that trained employees may subsequently leave for better jobs and hence increase turnover.

As mentioned above, 34 studies report on bundles combining skills-enhancing practices with motivation-enhancing practices. Eight studies combined skills-enhancing practices (e.g., training, staffing) with empowerment-enhancing practices (e.g., supervision, feedback, teamwork) [68, 73, 84, 86, 133, 134, 151, 152]. They mostly reported significant positive effects on organizational outcomes (e.g., quality of care and patient safety).

Among the skills-enhancing practices, training occurred most frequently (50 studies), followed by staffing and recruitment practices (23 studies). Most of these studies were associated with employee outcomes (e.g., retention, task performance, job satisfaction, and motivation), and less with organizational outcome (e.g., quality of care) (13 studies) and patient outcomes (2 studies). Only one study researched skills-enhancing training combined with motivation-enhancing practice (i.e., supplementary allowances) and showed improved employee outcomes (i.e., job satisfaction and reduced moonlighting) [79]. Two studies showed that written job descriptions (in combination with training, staffing, and empowerment- and motivation-enhancing practices) yielded significantly positive effects on organizational outcomes (i.e., efficiency and quality of care) [133, 168].

#### Empowerment-enhancing HRM practices

Empowerment-enhancing practices (*n* = 42, 49.4%) mainly entailed teamwork, communication, and supportive supervision. Only two studies considered purely empowerment-enhancing bundles, one of which showed improvements in the patient outcome maternal mortality [92], and the other reported improvement in the organizational outcomes quality of care and staff shortage [147].

Most studies (*n =* 30, 35.3%) that addressed empowerment-enhancing practices considered one empowerment-related practice combined with other practices. Eleven studies researched empowerment-enhancing practices (e.g., team work, supervision) combined with motivation-enhancing practices (e.g., compensation, scheduling) [70, 78, 99, 105, 110, 115, 117, 127, 136, 140, 163]. These studies mostly reported improvement on employee outcomes (e.g., satisfaction, retention, performance). Some reported improvement on organizational outcomes (e.g., quality of care, efficiency) and patient experience (i.e., satisfaction and clinical outcomes). However, one study [78] reported no improvement on the employee outcome turnover intention.

Communication/feedback practices (16 studies), teamwork (15 studies), and supervision (14 studies) occurred most frequently in combination with skills- and motivation-enhancing practices. Most of these studies were associated with improved employee satisfaction, motivation, retention, and performance. Nine studies reported improvement on the organizational outcomes (e.g., quality of care) and to a lesser extent to patient outcome [68, 73, 84, 86, 87, 91, 129, 133, 168].

Of the empowerment-enhancing practices, employee engagement, work-life balance, job autonomy, job security, and safety were less frequently researched (six studies). Of these studies, six reported positive effects and improvement on employee outcomes (e.g., job satisfaction, motivation, retention, task performance) [72, 117, 124, 132, 140, 169]. Positive significant effects on the organizational outcome quality of care were reported once [115].

## Conclusion

For the first time, an overview of studies that researched the link between HRM and performance in SSA hospitals is presented. The literature shows that HRM affects four different categories of performance outcomes: (individual) employee, team, organization (as a whole), and patient outcomes. Employee outcomes and organizational outcomes are frequently researched, whereas team outcomes and patient outcomes are significantly less researched. Evidence of the effect of HRM on patient outcomes, probably mediated via HRM outcomes, for now primarily builds on studies outside the SSA and studies with low quality of evidence within the SSA setting [17, 19, 24, 39]. Given the scarcity of human resources and the disproportional high burden of disease in SSA, further research on the effect of HRM practices on patient outcomes in SSA contexts is urgently called for. As previous studies reveal that contextual characteristics impact outcomes [16, 18, 33], contextual characteristics need to be taken into account, as can be attained by adopting the Context, Intervention, Mechanism, Outcome (CIMO) logic [52, 179, 180].

This review revealed 18 types of HRM practices that were researched in relation to performance of SSA hospitals. As shown in Table 7, this number is comparable to the 26 types of HRM practices presented by Boselie et al. [19]; 13 HRM practices (within high-performance work practice) shown by Combs et al. [20]; 10 HRM practices acknowledged by Hyde et al. [26]; and 6 HRM practices presented by Dieleman et al. [16]. Table 7 summarizes several reviews on HRM in different settings and shows that there is overlap in HRM practices. For example, training and education, compensation, recruitment, and team working are shown to be effective in many reviews. Although there is overlap in HRM practices researched in SSA context and the above mentions studies that researched HRM practices in a broader context (e.g., training, pay, and reward), three areas are under-explored in SSA. First, in SSA context, HRM practices related to employment are only researched in terms of staffing, rostering, and scheduling, but not in terms of selection, diversity, equal opportunity, exit management, and egalitarianism. On the other hand, employment regarding moonlighting is explored in SSA context, but rarely in overall HRM literature. Second, direct participation is studied in terms of communication, empowerment, and management, but not in terms of indirect participations through committees and councils, or in terms of socialization and social responsibility practices. Third, the professionalization of HRM function/department as a HRM practice is not researched at all in SSA context. These differences could be explained by the difficult SSA labor market that is characterized with low wages, the collectivistic and hierarchical organizational culture, and the lack of officially appointed HR functions.

**Table 7:** Overview of overall findings of systematic reviews on HRM and performance

Author (year)	Aim of review	No.	Setting	HRM practices	Summary of findings
This review	To present a systematic review of empirical studies investigating the relationship between HRM and performance in SSA hospitals.	111	Saharan Africa Hospitals	18 HR practices: -Training and education -Task delegation/task shifting -Compensation, salary, incentives -Promotion/recognition -Scheduling and rostering -Management/leadership support -Team work -Performance appraisal -Feedback/communication -Staffing -Selection/recruitment -Mentorship -Employee engagement -WLB -Job autonomy -Job security/safety -Written job description	HRM practices in SSA are linked to all categories of performance outcomes: individual employee outcomes (task performance, job satisfaction, motivation, retention, reduction in workload and moonlighting); team outcomes, organizational performance outcomes (quality of care, patient safety, timeliness, service efficiency, staff shortage) and patient outcomes (patient experience and clinical outcomes).
Hyde et al. (2006)	To investigate how HRM can influence performance in organizations by addressing the question “How can HRM help NHS organizations to achieve their goals?”	97	European Hospitals	10 HRM practices: -Training -Pay -Involvement -Selection -Team working -Performance appraisal -Job security -Job design -Equal opportunities -Career development	Bundles of practices are more likely to positively affect performance than single practices. There is insufficient evidence that a specific HRM practice is superior in increasing performance. Local and wider external contextual factors need to be taken into account when doing research in health sector.
Boselie et al. (2005)	To see whether there might be commonalities and widely accepted trends in the theoretical perspectives, conceptualizations and methodologies used in the field of HRM and performance research.	104	European (Dutch) hospitals	26 HR practices: -Training -Contingent pay and rewards, -Performance management -Recruitment -Team working -Direct participation -Good wages -Communication -Internal promotion -Job design -Autonomy -Employment security -Benefits -Formal procedures -HR planning -Financial participation -Symbolic egalitarianism -Attitude survey -Indirect participation -Diversity and equal opportunities -Job analysis -Socialization -Family-friendly policies -Exit management -Effectiveness of HR function -Social responsibility practices	The relationship between (some form of) HRM intervention and (some indicator of) performance is mediated by linking mechanisms.
Combs et al. (2006)	To identify and analyze studies that investigate the relationship between at least one HPWP and organizational performance.	92	Manufacturing and service organizations	13 HRM practices within HPWP: -Incentive compensation -Training -Compensation level -Participation -Selectivity -Internal promotion -HR planning -Flexible work -Performance appraisal -Grievance procedures -Teams -Information sharing -Employment security	HPWPs have a higher impact than individual practices on organizational performance (focused on operational and financial performance outcomes).
Dieleman et al. (2009)	to explore if realist review of published primary research provides better insight into the functioning of HRM interventions	48	Low- and middle-income countries	6 HRM practices: -Continuing education -Supervision -Payment of incentives -Decentralization of HRM functions -Regulation -Combination of HR practice such as training	HRM interventions can improve health workers’ performance. Mechanisms such as increased knowledge and skills, feeling obliged to change and health workers’ motivation caused change. Continuing education is likely to be effective in short term. Combined interventions are more likely to be effective in the long term. Thereby, context should be taken into account.

The minority of included studies focused on single HRM practices. They mostly found positive effects on performance. Most included studies reported on implementation of HRM bundles, as is in line with Subramony [28] and Boselie et al. who claim that HRM bundles are likely to be synergistic, thus yielding stronger effects on performance than single HRM practices [19].

### Single HRM practices versus HRM bundles

In SSA, training and education are the most researched single HRM practice. Training is one of seven Pfeffer’s best practices, which is believed to lead to superior outcomes in any setting [181]. Training is evidence to positively impact outcomes in all four performance categories. Training and educating caregivers in non-technical skills (e.g., communication, awareness, interaction) is a worldwide trend within the hospital setting and is proven to lead to higher team performance, patient safety, and organizational performance [181–183]. Task shifting/ role delegation in SSA hospitals is the second most researched single HRM practice, and mostly evidenced to relate to improved organizational and patient outcomes [95, 103, 161, 162, 164]. Task shifting is seen as one of the most important policy options to alleviate workforce shortage and skill mix imbalances in low-resource countries [184]. The most common task shifting, which requires leadership support, takes place in HIV treatment where tasks are delegated from doctors to nurses and other non-physician clinicians [185, 186].

Most included studies researched HRM bundles that included practices from multiple HRM domains: motivation-enhancing, skills-enhancing, and empowerment-enhancing [28]. Motivation-enhancing practices were most frequently researched within HRM bundles, followed by skills-enhancing practices and empowerment-enhancing practices. A significant amount of studies provided evidences on the link between a HRM bundle and improved performance (e.g., [16, 17, 19–21, 24]). Our findings show that an improvement in a specific outcome measure can be accomplished by different HRM practices or bundles and that similar HRM practices or bundles could enhance different outcome measures. For instance, job satisfaction could be improved through (a combination of) single HRM practices or bundles regarding training, management support, teamwork, promotion, autonomy, financial incentives, scheduling, and performance appraisal (e.g., [70, 79, 98, 109, 112, 117, 123, 127, 136]). This also holds for the outcomes; retention, motivation, and quality of care. Eight studies that examined a similar HRM bundles reported improvement in diverse outcome measures (e.g., employee performance, organizational outcome such as quality of care, efficiency, and patient satisfaction) [81, 82, 87, 91, 137, 176, 178].

Previous studies have shown the importance of an internal fit within a HRM bundle, referring to an alignment between HRM practices [24, 26, 29, 187]. Notable is that several included studies have combined teamwork with individual financial incentives such as salary (e.g., [99, 101, 105, 129, 135]). Although this combination of HRM practices is in HRM literature often labelled as “deadly”, financial incentives have shown to be effective and desirable in improvement programs in SSA [9, 34, 36, 37, 49–51]. This calls for further research.

### Limitations and future recommendations

Our study included evidence on relationships between HRM practices and hospital performance in 19 SSA countries. Given the variety in results, we call for caution when generalizing the results to all SSA countries, or to health centers and clinics in SSA. Recognizing the importance of tailoring interventions to both internal and external context (also referred to as the “best fit approach” of HRM [27, 44, 45, 188]), we recommend future empirical research to report on relevant internal and external contextual factors. This will enable to build evidence on the mechanisms explaining how context and interventions together produce outcomes, as opposed to developing an evidence base for all of the different SSA contexts. Second, this review was restricted to peer-reviewed English articles and did not including books, grey literature, or any documents published in a foreign language. As a result, we may have failed to identify some evidence. Additionally, the inclusion criteria may have induced bias towards effective implementations and caused us to exclude interventions which produced little or adverse performance effects. Lastly, we note that our review produced little evidence on a direct relationship between HRM interventions and patient outcomes, or on outcomes at a team level. We recommend to conduct research in these areas, as team performance is evidenced to be particularly related to patient outcomes of hospitals [189, 190].

## Additional files

Additional file 1: Search terms. (DOCX 16 kb)

Additional file 2: Number of studies that link a specific HRM practice to a specific outcome. (XLSX 11 kb)

Additional file 3: SSA countries represented in selected studies. (DOCX 14 kb)
