# Supplementary material for: The effect of human resource management on performance in hospitals in Sub-Saharan Africa: a systematic literature review
Source: Hum Resour Health. 2018 Aug 2;16:34. doi: 10.1186/s12960-018-0298-4 (PMC6090989; doi:10.1186/s12960-018-0298-4)
Supplement: Supplementary file 1 — Search terms. (DOCX 16 kb) [file 12960_2018_298_MOESM1_ESM.docx]

Additional file 1 Search Terms

| **Category 1: geographical setting** | **Category 2: health care setting** | **Category 3: topic** |
| --- | --- | --- |
| Sub-Saharan Africa, Benin, Malawi, Ethiopia, Kenya, South Africa, Nigeria, Tanzania, Ghana, Uganda, Mali, Burkina Faso, Namibia, Rwanda, Eritrea, Lesotho, Zimbabwe, Burundi, Mozambique, Senegal, Botswana, Zambia, Angola, Cameroon, Cape Verde, Central African Republic, Chad, Comoros, Congo (Brazzaville), Congo (Democratic Republic), Côte d'Ivoire, Djibouti, Equatorial Guinea, Gabon, The Gambia, Guinea, Guinea-Bissau, Liberia, Madagascar, Mauritania, Mauritius, Niger, Sao Tome and Principe, Seychelles, Sierra Leone, Somalia, Sudan, South Sudan, Swaziland, Togo | hospital(s)  health professional  health worker  employee  health workforce  health care organization(s)  health services organization(s)  health facility(ies) | human resource(s) management / practice |
|  |  | human resource for health |
|  |  | employee engagement |
|  |  | staffing/recruitment |
|  |  | retention |
|  |  | training |
|  |  | incentives |
|  |  | compensation |
|  |  | employee performance |
|  |  | motivation |
|  |  | job satisfaction |
|  |  | performance management |
|  |  | recognition |
|  |  | turnover intention |
|  |  | teamwork |
|  |  | supervision |
|  |  | communication |
|  |  | promotion |
|  |  | scheduling |
|  |  | feedback |

**Illustration: the search strategy for Embase.com**

('personnel management'/exp OR ((leadership/de OR 'organization and management'/de OR 'competence'/de) AND (staff/de OR 'health care personnel'/de OR 'nursing staff'/de OR manpower/de OR 'health care manpower'/de )) OR 'nursing competence'/de OR 'professional competence'/de OR (((personnel* OR employe* OR hr OR worker* OR healthworker* OR nurs* OR doctor* OR physician* OR workforce* OR midwife* OR staff* OR ward* OR manpower) NEAR/3 (management* OR schedul* OR managing OR motivat* OR leadership* OR empower* OR needs OR recruit* OR retention* OR skills OR competenc)) OR (Human NEAR/3 resourc*) OR hrm ):ab,ti) AND ('hospital'/exp OR 'hospital management'/exp OR 'hospital organization'/exp OR 'hospital personnel'/exp OR (hospital* OR ((care OR healthcare) NEXT/1 (facilit* OR organization*))):ab,ti) AND ('Africa south of the Sahara'/exp OR 'African'/de OR 'Central African'/exp OR 'East African'/exp OR 'Namibian'/de OR 'West African'/exp OR (((south OR sub OR west*) NEAR/3 sahara*) OR (africa NEAR/3 (south* OR west* OR east* OR central)) OR Angol* OR Cameroon* OR Chad OR chadese OR Congo* OR Equatorial-Guinea* OR Gabon* OR Sudan* OR Zambia* OR Burundi* OR Comoros* OR Djibout* OR Eritrea* OR Ethiopia* OR Kenya* OR Madagascar* OR Malawi* OR Mauriti* OR Mayotte* OR Mozambiqu* OR Reunion* OR Rwand* OR Seychell* OR Somali* OR Tanzania* OR Ugand* OR Benin* OR Burkina-Faso* OR Cote-d-Ivoire* OR Ivory-Coast* OR Gambia* OR Ghana OR ghanese OR (Guinea* NOT Guinea-pig*) OR Liberia* OR Mali OR malinese OR Mauritania* OR Niger* OR Saint-Helen* OR Sao-tome* OR Senegal* OR Sierra-Leon* OR Togo OR togolese OR Zimbabw* OR botswan* OR burund* OR cape-verd* OR lesotho* OR namibi* OR seychele* OR swaziland*)) NOT ([Conference Abstract]/lim OR [Letter]/lim OR [Note]/lim OR [Editorial]/lim)
