# Supplementary material for: The effect of human resource management on performance in hospitals in Sub-Saharan Africa: a systematic literature review
Source: Hum Resour Health. 2018 Aug 2;16:34. doi: 10.1186/s12960-018-0298-4 (PMC6090989; doi:10.1186/s12960-018-0298-4)
Supplement: Supplementary file 3 — SSA countries represented in selected studies. (DOCX 14 kb) [file 12960_2018_298_MOESM3_ESM.docx]

Additional file 3 SSA Countries represented in selected studies

|  | **Country** | **# of studies** |
| --- | --- | --- |
| 1 | S. Africa | 32 |
| 2 | Tanzania | 14 |
| 3 | Kenya | 13 |
| 4 | Nigeria | 10 |
| 5 | Ethiopia | 8 |
| 6 | Uganda | 8 |
| 7 | Ghana | 6 |
| 8 | Malawi | 6 |
| 9 | Rwanda | 4 |
| 10 | Mali | 3 |
| 11 | Mozambique | 3 |
| 12 | Zambia | 3 |
| 13 | Namibia | 2 |
| 14 | Benin | 2 |
| 15 | Senegal | 2 |
| 16 | Burundi | 1 |
| 17 | Mauritious | 1 |
| 18 | Botswana | 1 |
| 19 | Zimbabwe | 1 |

Note that the number of studies exceed the total number of 111 studies as studies conducted research in multiple countries
